# Lateral flow immunoassay-based laboratory algorithm for rapid diagnosis of diphtheria

**DOI:** 10.12688/openreseurope.15038.1

**Published:** 2023-04-25

**Authors:** Vyacheslav G. Melnikov, Anja Berger, Alexandra Dangel, Andreas Sing

**Affiliations:** 1 National Conciliary Laboratory on Diphtheria, Bavarian Health and Food Safety Authority, Oberschleißheim, 85764, Germany; 2 Public Health Microbiology, National Conciliary Laboratory on Diphtheria, Bavarian Health and Food Safety Authority, Oberschleißheim, 85764, Germany

**Keywords:** Corynebacterium diphtheriae; Corynebacterium ulcerans; LFIA; simulated diphtheria specimens; diphtheria laboratory diagnosis

## Abstract

**Background: **In industrialised countries diphtheria is a rare but still life-threatening disease with a recent increase in cases due to migration and zoonotic aspects. Due to the rarity of the disease, laboratory diagnosis of diphtheria is often carried out in central reference laboratories and involves the use of sophisticated equipment and specially trained personnel. The result of the diphtheria agent detection can usually be obtained after 5-6 days or more. Authors suggest a Lateral Flow Immunoassay (LFIA)-based laboratory algorithm for the diagnosis of diphtheria, which may render less time in issuing a result and could promote the testing be performed in laboratories closer to the patient.

**Methods: **LFIA for diphtheria toxin (DT) detection was designed using a pair of monoclonal antibodies to receptor-binding subunit B of the DT, and validated with 322 corynebacterial cultures as well as 360 simulated diphtheria specimens. Simulated diphtheria specimens were obtained by spiking of human pharyngeal samples with test strains of corynebacteria. The simulated specimens were plated on selective tellurite agar and after 18-24 hours of incubation, grey/black colonies characteristic of the diphtheria corynebacteria were examined for the DT using LFIA.

**Results: **The diagnostic sensitivity of the LFIA for DT detection on bacterial cultures was 99.35%, and the specificity was 100%. Also, the LFIA was positive for all pharyngeal samples with toxigenic strains and negative for all samples with non-toxigenic strains. For setting LFIA, a 6-hour culture on Elek broth was used; thus, under routine conditions, the causative agent of diphtheria could be detected within two working days after plating of the clinical specimen on the tellurite medium of primary inoculation.

**Conclusions: **The availability of such a simple and reliable methodology will speed up and increase the accuracy of diphtheria diagnosis globally

## Plain language summary

Before the introduction of vaccine, diphtheria was a leading cause of childhood death around the world. With the evidenced rise in cases, it seems that diphtheria is a re-emerging old foe of humankind and could become a potential major global threat. The current diphtheria diagnostic methodology is complex and time-consuming. Considering this, scientists of the EU-funded ‘DIFTERIA’ project have raised the issue of a reliable diagnostic method to be prepared for possible outbreaks. A lateral flow immunoassay (LFIA) has been developed that detects diphtheria toxin directly in a culture of bacteria grown on the selective agar for primary inoculation in as little as six hours. Thus, the time for diagnosis of diphtheria will decrease from 5–6 days or more, given the need to deliver a sample from the patient to the central reference laboratory, to 2 days. Implementation of this novel accelerated and simplified method will improve the accuracy of the diphtheria diagnosis in Europe and worldwide, enabling the timely initiation of treatment and preventing the pathogen from capturing new territories.

## Introduction

During the 1990s in Russia and neighboring countries the epidemic of diphtheria affected about 200,000 people with 5000 deaths. It was the largest diphtheria epidemic since the 1950s, when diphtheria immunization campaigns began ^1^. The emergence of the epidemic was facilitated by the displacement of significant masses of people caused by the collapse of the USSR, and the associated decline in living standards, the quality of medical care and the public health infrastructure. At present, diphtheria is still a widespread infectious disease in the world. In India more than 100,000 diphtheria cases have been registered since 2000. Ethiopia reported 12451 cases in 2019–2020, Nigeria – 4159 in 2018–2019 ^2^. Due to ethnic, confessional conflicts and humanitarian crises outbreaks of the infection occurred in Bangladesh, Yemen, Venezuela in 2017–2019 ^3– 5^. Increasing global travel and the relocation of people is contributing to the spread of diphtheria to areas where it has not been seen for many years ^6^. Diphtheria outbreaks have been recently registered in Europe among refugees from diphtheria-endemic regions of Asia and Africa ^7, 8^. Toxigenic *Corynebacterium diphtheriae* and *C. ulcerans* pose a serious risk for people with low level of immune protection, the number of which has increased worldwide due to low adult re-vaccination rate, vaccine hesitancy and the coronavirus disease 2019 (COVID-19) pandemic’s backsliding on childhood vaccinations ^9^.

It is well acknowledged that the earlier diphtheria is diagnosed, the more favorable the outcome of the disease can be expected and the earlier the epidemiological surveillance service can be warned. The purpose of laboratory diagnosis of diphtheria is to indicate the presence in the clinical sample of corynebacteria that produce diphtheria toxin (DT). The current diagnostic methodology is complex and time-consuming. The WHO Manual ^10^ suggests the following steps for laboratory diagnosis of diphtheria: isolation of a pure culture, biochemical identification of the suspected diphtheria agent and then testing of the identified isolate for the ability to produce DT. Usually, in the “real world” it takes 5–6 days to complete the entire diagnostic cycle, or even more, given the time of transportation of the purified suspicious culture to the reference laboratory. It is obvious that the use of this "classic" microbiological approach in diphtheria outbreaks is inappropriate due to its length and organizational complexity. Due to the need to quickly study a large number of clinical samples, an important task is to speed up the procedure for detecting the diphtheria agent in clinical material, as well as to simplify the diagnostic methodology so that it can be carried out even in peripheral bacteriological laboratories.

The main element in the diphtheria diagnostic scheme is the detection of DT, the major virulence factor in pathogenic corynebacteria. The gold standard method for detecting the production of DT by corynebacteria, reaction of immunoprecipitation in agar or Elek test, a complicated technology requiring time, the availability of proven reagents and special knowledge, is performed mainly by specialized reference laboratories ^10^. Polymerase chain reaction (PCR) cannot serve as an alternative to the Elek test, since it does not detect DT itself, but its gene, while non-toxigenic *tox* gene-bearing corynebacteria (NTTB) circulate worldwide ^10– 12^.

To determine the presence and the level of DT production by *C. diphtheriae* and *C. ulcerans*, an immunoassay based on monoclonal antibodies (mAbs) has been designed ^13^. A pair of mAbs specific to subunit B of DT was developed, which made it possible to detect DT in a sandwich enzyme-linked immunosorbent assay (ELISA) with a detection limit of DT less than 1 ng/mL. The mAbs used in the ELISA proved to be quite discriminatory, so in this study we used them for the design of the Lateral Flow Immunoassay (LFIA), a method that can reduce the labor and cost of laboratory diagnosis of diphtheria. The diagnostic sensitivity and specificity of the LFIA were determined using a collection of diphtheria group (toxigenic and non-toxigenic) and non-diphtheria corynebacteria. Besides, we investigated the value of an accelerated diagnostic approach ^14^ that differs from the classical algorithm. It includes the assessment of DT production in bacterial colonies grown on selective tellurite plate of primary inoculation, prior to the stage of isolation and identification of a pure culture. This approach allows much earlier indication of the presence or absence of toxigenic corynebacteria in a patient and thereby confirms the diagnosis of diphtheria or makes it less likely.

## Methods

### Preparation of the lateral flow test

LFIA test strips were manufactured by Senova, Weimar, Germany, under the H2020-MSCA-IF-2018 (843405-DIFTERIA) research program using reagents previously used to develop ELISA for DT detection ^13^. These included polyclonal goat anti-mouse IgG (control Ab) and a pair of mouse monoclonal antibodies to the diphtheria toxin B subunit (both IgG _1_): anti-DT 675-3 (capture mAb) and anti-DT 676-3 labeled with colloidal gold (detection mAb). The test strips were placed into lateral flow housings and were sealed with a desiccant in airtight foil pouches. When in foil pouches, the LFIA cassettes were stable for at least 24 months at 4°C and at RT. A detection limit for DT (Sigma Aldrich) in Elek broth ^15^ was 0.3±0.1 ng/ml.

### LFIA validation

*Strains tested*. 322 strains were studied by the LFIA: 305 strains of diphtheria group corynebacteria ( *C. diphtheriae, C. ulcerans, C. silvaticum, C. pseudotuberculosis*) - 154 toxigenic, 151 nontoxigenic, and 17 of non-diphtheria nasopharyngeal corynebacteria ( *C. pseudodiphtheriticum, C. striatum, C. amycolatum, C. accolens, C. tuberculostearicum*), all obtained from the German Conciliary Laboratory on Diphtheria (GCLoD) culture collection ( Table 1). Strains were both of human and animal origin and isolated in Germany in 2011-2022. They were identified at the GCLoD by culture, biochemical differentiation (API Coryne, bioMèrieux, Nürtingen, Germany), MALDI-TOF analysis (MALDI Biotyper; Bruker Daltonics, Bremen, Germany), and the presence of the *tox* gene was determined by real-time PCR ^10^; DT was detected by the optimised Elek test ^16^ using purified diagnostic diphtheria antitoxin (Microgen, Moscow, Russia). 24-hour cultures of corynebacteria on Columbia Blood Agar (Oxoid) were inoculated on Elek broth ^15^, and after 6 h of cultivation at 37°C, 100 µL of the liquid culture was applied to the LFIA cassette, and the result was recorded after 15 minutes. All the experiments were conducted in triplicate.

**Table 1.** Results of the cystinase test and comparison of the Elek test and LFIA for the detection of diphtheria toxin (DT) in 322 strains of diphtheria and non-diphtheria corynebacteria.

Species and gene *tox* status	No. of strains tested	Pisu test	DT by Elek test	DT by LFIA
Diphtheria group corynebacteria				
*C. diphtheriae tox+*	46	+	+	+
*C. diphtheriae tox*-	76	+	-	-
*C. diphtheriae* belfanti *tox*-	12	+	-	-
*C. diphtheriae* (NTTB *) *tox+*	3	+	-	-
*C. ulcerans tox+*	107	+	+	+
***C. ulcerans KL 1902 tox+***	**1**	**+**	**+**	**-**
*C. ulcerans tox*-	46	+	-	-
*C. silvaticum* (NTTB *) *tox+*	4	+	-	-
*C. pseudotuberculosis tox*-	10	+	-	-
Non-diphtheria corynebacteria **				
*C. pseudodiphtheriticum*	3	-	-	-
*C. striatum*	6	-	-	-
*C. amycolatum*	5	-	-	-
*C. accolens*	2	-	-	-
*C. tuberculostearicum*	1	-	-	-

*NTTB – non-toxigenic *tox*-bearing strains. **Non-diphtheria corynebacteria never possess the gene *tox*. Highlighted in bold – the strain *C. ulcerans* KL 1902 with “unusual” properties.

*Simulated diphtheria specimens.* Eight corynebacterial strains isolated from clinical samples and collected by the GCLoD: toxigenic *C. diphtheriae* biovar gravis KL 950; non-toxigenic *tox* gene negative *C. diphtheriae* biovar gravis KL 1749; toxigenic *C. diphtheriae* biovar mitis KL 1755; non-toxigenic *tox* gene positive *C. diphtheriae* biovar mitis KL 1810 (NTTB); toxigenic *C. ulcerans* KL 1819; weakly toxigenic *C. ulcerans* KL 568 and KL 1779, *C. pseudodiphtheriticum* KL 1452 (resident nasopharyngeal corynebacteria) as well as three reference *C. diphtheriae* strains (toxigenic biovar gravis NCTC 10648, weakly toxigenic biovar gravis NCTC 3984 and non-toxigenic *tox* gene negative biovar belfanti NCTC 10356) and one reference *C. ulcerans* non-toxigenic *tox* gene negative strain NCTC 12077 were included in this study. Cultures were grown on Columbia Blood Agar (Oxoid) for 18-24 h before use. 

For spiking material, 120 severe acute respiratory syndrome coronavirus 2 (SARS-CoV-2) negative human pharyngeal samples delivered to the Bavarian Health and Food Safety Authority, Oberschleissheim, Germany for SARS-CoV-2-testing were used. Samples were transported in screw cap plastic tubes with Amies liquid medium containing no antibiotics (ESwabs, Copan Diagnostics, CA, USA). The simulated diphtheria specimens were prepared by spiking of the 120 pharyngeal samples with the 12 corynebacterial test strains mentioned above (1 strain per 10 samples, Table 2). The optical density of an initial suspension of the bacterial inoculum (10^9 cells/ml) was adjusted using a Densimat (bioMèrieux). Three dilutions (10^5, 10^6 and 10^7 cells per mL) of each corynebacterial strain suspension were prepared in PBS. Ten μl of each dilution of corynebacteria was added to 1 ml of the pharyngeal sample in Amies transport medium, and 25 μl of this simulated diphtheria specimen was streaked onto selective BD Hoyle’s Tellurite Agar (Becton Dickinson) to obtain isolated colony growth. Prior to contamination with corynebacteria, each pharyngeal specimen was plated on blood agar and on Hoyle's tellurite agar. The presence of growth of the pharyngeal microbiota on blood agar indicated that the clinical sample was taken and transported correctly. The lack of growth of colonies of diphtheria corynebacteria on the tellurite agar demonstrated that initially the individual was not a diphtheria carrier. Totally 360 simulated diphtheria specimens were studied (120 pharyngeal samples, each containing 10^3, 10^4 or 10^5 cells/ml of test culture, respectively). After plating the simulated diphtheria specimens, growth on Hoyle’s tellurite agar was examined after 18–24 hours of incubation at 37°C, and a mixture of 5–10 grey/black uniform colonies, suspicious for corynebacteria, were inoculated on Elek broth ^15^ for LFIA. To set up the reaction, 100 μl of a 6-hour Elek broth culture, was applied to the LFIA.

**Table 2.** Results of a study of grey/black colonies grown on Hoyle’s tellurite agar after plating of diphtheria simulated specimens using the Pisu test and LFIA.

No	Test strains used for diphtheria simulated specimens design and their *tox*/Tox status *	No. of human pharyngeal samples	Pisu test	DT by LFIA
1	NCTC 10648 *C. diphtheriae* gravis *tox*+ Tox+	10 samples	+	+
2	NCTC 10356 *C. diphtheriae* belfanti *tox*- Tox-	10 samples	+	-
3	NCTC 3984 *C. diphtheriae* gravis *tox*+ Tox+	10 samples	+	+
4	NCTC 12077 *C. ulcerans* *tox*- Tox-	10 samples	+	-
5	KL 950 *C. diphtheriae* gravis *tox*+ Tox+	10 samples	+	+
6	KL 1749 *C. diphtheriae* gravis *tox*- Tox-	10 samples	+	-
7	KL 1755 *C. diphtheriae* mitis *tox*+ Tox+	10 samples	+	+
8	KL 1810 *C. diphtheriae* mitis *tox*+ Tox- (NTTB)	10 samples	+	-
9	KL 568 *C. ulcerans* *tox*+ Tox+	10 samples	+	+
10	KL 1779 *C. ulcerans* *tox*+ Tox+	10 samples	+	+
11	KL 1819 *C. ulcerans* *tox*+ Tox+	10 samples	+	+
12	KL 1452 *C. pseudodiphtheriticum* *tox*- Tox-	10 samples	-	-

*These initial data were defined at the German Conciliary Laboratory on Diphtheria.

A cystinase assay (Pisu) targeting diphtheria corynebacteria was used to confirm their growth on Hoyle's tellurite agar after plating the simulated specimens. Along with the LFIA sampling, a mixture of the bacterial colonies grown on Hoyle’s primary inoculation plate was stabbed into the semi-solid Pisu Modified Medium (SRCAMB, Obolensk, Russia). In order to test how well the Pisu assay works with corynebacteria isolated in places other than Russia, it was also run with all 322 strains used for the LFIA validation. The cystinase assay was conducted according to the manufacturer’s instructions. With a positive cystinase test, the Pisu medium turns black, which is associated with the formation of bismuth sulfide during the interaction of the bismuth citrate component of the medium with hydrogen sulfide, appearing after the enzymatic cleavage of L-cystine by diphtheria corynebacteria. The test result is recorded in 6 hours of cultivation at 37°C. After 18–24 hours of cultivation, the result of the Pisu test (as well as LFIA) is not lost, but becomes even more pronounced. The advantage of the Pisu Modified Medium is that it does not require autoclaving and the addition of animal serum.

For a detailed look at the DT sequence of *C. ulcerans* KL1902., whole genome sequencing (WGS) was performed with Illumina NextSeq550 sequencing with 2x150bp paired-end reads (Illumina, San Diego, CA, USA), after DNA isolation on the Promega Maxwell system (Promega, Mannheim, Germany) and library preparation with the Illumina DNA prep kit (Illumina, San Diego, CA, USA). Bioinformatics analyses including raw data QC, genome assembly, assembly QC and extraction of *tox* and DT sequences from the assemblies were done as described in 17.

## Results

### Validation of the LFIA using corynebacteria strains

**Figure 1.**
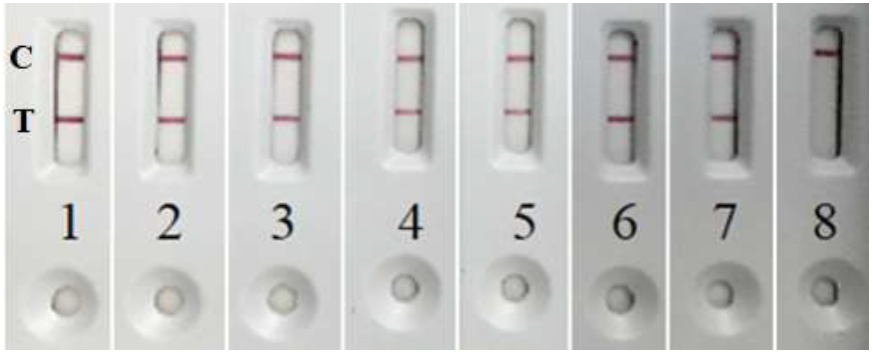
Positive LFIA with toxin producing *C. diphtheriae* strains: 1) KL 1670; 2) KL 1675; 3) KL 1686; 4) KL 1707; 5) KL 1747; 6) KL 1754; 7) KL 1755; and negative LFIA with non-toxigenic *C. diphtheriae* strain: 8) KL 1810 (NTTB).

As shown in Table 1 and Figure 1, the LFIA designed on a pair of the mAbs to subunit B of the DT, detected DT in corynebacterial cultures with high accuracy. For 321 of the 322 strains of corynebacteria studied by the LFIA, the results were consistent with those of the Elek test. Of the 154 strains of *C. diphtheriae* and *C. ulcerans* producing DT in the Elek test, 153 were positive in the LFIA. Gene *tox* negative diphtheria corynebacteria – *C. diphtheriae*, *C. ulcerans* and *C. pseudotuberculosis* (144 strains), as well as 17 cultures of non-diphtheria corynebacteria were LFIA negative. Gene *tox* positive but not expressing DT *C. diphtheriae* and *C. silvaticum* (seven NTTB strains) were also LFIA negative.

**Figure 2.**
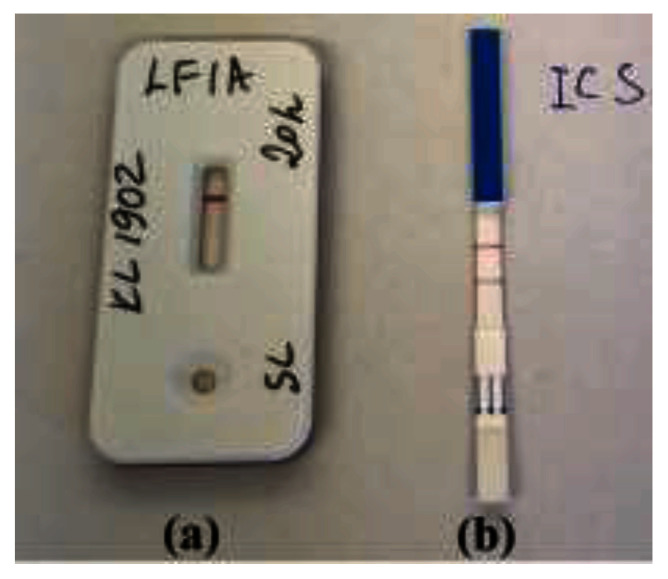
Results of the diphtheria toxin (DT) detection in “unusual” strain *C. ulcerans* KL 1902 by LFIA and Immunochromatographic Strip (ICS) test: **(a)** negative LFIA targeting subunit B of the DT and **(b)** positive ICS test targeting subunit A of the DT.

The only discrepancy was the *tox* gene positive (by PCR) and DT producing (by Elek test) strain *C. ulcerans* KL 1902, which was LFIA negative. To better understand the discrepancy, the toxigenicity status of strain KL 1902 was studied by additional methods. This strain was found to be negative in ELISA ^13^ based on the same mAbs as LFIA. We also used the immunochromatographic strip (ICS) test ^18^. The ICS test was designed on the mAb against subunit A (catalytic domain) of DT as detecting antibody and polyclonal diphtheria antitoxin as capture antibody. Therefore, ICS test catches DT by the fragment A, while the LFIA/ELISA – by the fragment B of the DT. As shown in Figure 2, ICS test was positive, which confirms DT production by the *C. ulcerans* KL 1902 strain.

We performed whole genome sequencing for *C. ulcerans* KL 1902. WGS raw data and tox gene sequence are available at the National Center of Biotechnology Information (NCBI) ^19– 21^. It was found that there is a single nucleotide polymorphism (SNP: *tox*:C1238T, changing triplet CCA to CTA), leading to amino acid change from Prolin to Leucin in position 413 [(P413L) in the B-subunit/R-domain]. This SNP is absent from the other *C. ulcerans* or *C. diphtheriae* toxin gene sequences. It might be speculated that this unique mutation in the DT gene (P413L) may disrupt the folding of the toxin molecule and thereby prevent mAbs from binding to the toxin molecule in our LFIA/ELISA. However, since such a mutated *C. ulcerans* strain with unusual properties was found only once among a large group of diphtheria corynebacteria studied by us, the LFIA methodology for detecting DT should be regarded as highly specific and can be recommended for practical use.

### Validation of the LFIA using simulated diphtheria specimens

Our attempts to use an Elek broth as a liquid medium for primary inoculation of simulated diphtheria specimens in order to accumulate the DT turned out to be ineffective even with a longer incubation time (24 hours). The associated pharyngeal microbiota inhibited the growth and DT production of the diphtheria agent in liquid Elek medium. Chemical additives known to suppress the pharyngeal microbiota other than corynebacteria (potassium tellurite, fosfomycin, 8-oxyquinoline sulfate) did not increase DT production (data not shown). It was concluded that the pathogen can be reliably detected only when using the traditional method of cultivation on solid media. So, in this study, selective tellurite agar was used to obtain the growth of test strains of corynebacteria from simulated diphtheria specimens.

Comparison of selective tellurite agars: a) Columbia Blood Agar Base (Oxoid) supplemented with 7% sheep blood and 0.035% potassium tellurite; b) Korinebakagar (SCRAMB, Obolensk, Russia), which contains a hemophilic bacteria growth stimulator used as a blood substitute, with 0.025% potassium tellurite, and c) ready-to-use BD Tellurite Agar (Hoyle) in Petri dishes showed a significant advantage of the Hoyle’s medium in terms of germination and growth rate of test strains of corynebacteria. A comparable number of colonies grew after inoculation of corynebacteria on Hoyle’s tellurite agar and Columbia blood agar (data not shown). Therefore, Hoyle’s medium was used in further experiments.

**Figure 3.**
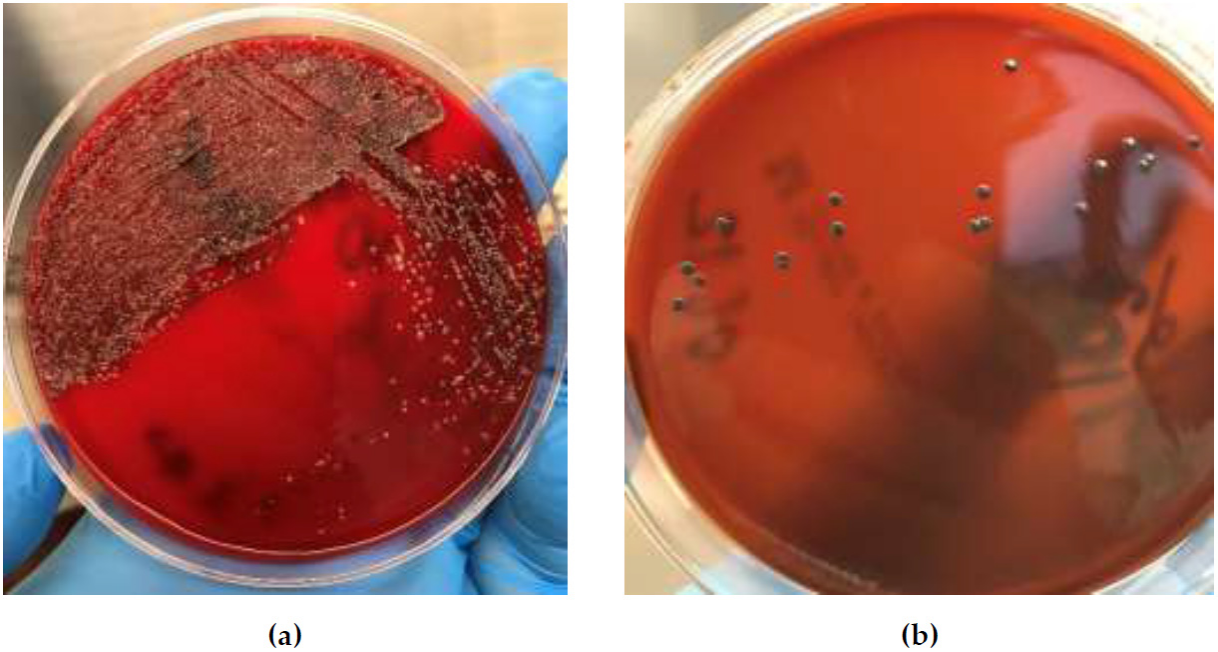
Colonies on Columbia Blood Agar ( **a**) and Hoyle’s tellurite agar ( **b**) after plating the same pharyngeal microbiota sample spiked with 10 ^3^/ml *C. diphtheriae* NCTC 3984 (simulated diphtheria specimen).

When inoculating simulated diphtheria specimens on Hoyle’s tellurite plates, single colonies grew from corynebacterial culture dilution 10 ^3^/ml, dozens of colonies – from dilution 10 ^4^/ml, and hundreds of colonies – from dilution 10 ^5^/ml. Test strains of corynebacteria from the simulated diphtheria specimens grew on Hoyle’s tellurite agar in nearly pure culture, which demonstrated the excellent properties of this selective medium in supporting the growth of the target group of corynebacteria, as well as inhibiting the growth of the concomitant pharyngeal microbiota ( Figure 3). It is known that both toxigenic and non-toxigenic corynebacteria can be present in a clinical specimen from a single patient, so multiple colonies should be tested for toxigenicity. A mixture of 5–10 uniform grey/black smooth, opaque, soft buttery colonies with the entire edge, 1–3 mm in size, grown on tellurite agar after 18–24-hour incubation, was used. A 1.5 ml Eppendorf tube with 0.5 ml Elek broth was inoculated with this mixture for the DT testing by LFIA. The culture was smeared on the inner wall of the tube and suspended in medium. The remainder of the colony mixture sample was stabbed into the Pisu medium. For each of these tests, even a minimal amount of culture from tellurite agar was sufficient in the form of a small black coating on the tip of a plastic inoculation needle.

**Figure 4.**
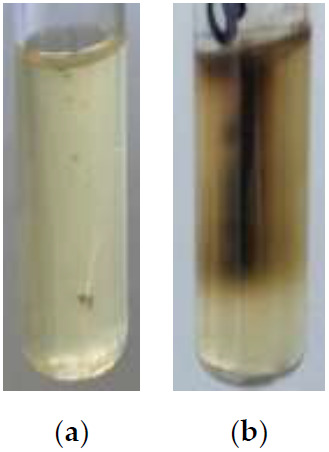
Pisu test tubes inoculated with *C. pseudodiphtheriticum* KL 1452 - cystinase negative culture ( **a**) and *C. diphtheriae* NCTC 3984 – cystinase positive culture ( **b**) grown on Hoyle’s tellurite agar after plating of simulated diphtheria specimens.

The Pisu test has been found to be suitable for distinguishing cystinase-positive diphtheria group corynebacteria both in purified cultures and in primary cultures grown from simulated samples. All 305 strains of the diphtheria group ( *C. diphtheriae, C. ulcerans, C. silvaticum, C. pseudotuberculosis*) were, as expected, Pisu positive, and 17 strains of non-diphtheria corynebacteria ( *C. pseudodiphtheriticum, C. striatum, C. amycolatum, C. accolens, C. tuberculostearicum*) were Pisu negative ( Table 1). When plating simulated diphtheria specimens with toxigenic or non-toxigenic *C. diphtheriae* and *C. ulcerans*, the cultures primarily grown on Hoyle’s agar were cystinase positive, while the Hoyle’s cultures derived from *C. pseudodiphtheriticum*-loaded specimens were Pisu negative ( Figure 4, Table 2).

**Figure 5.**
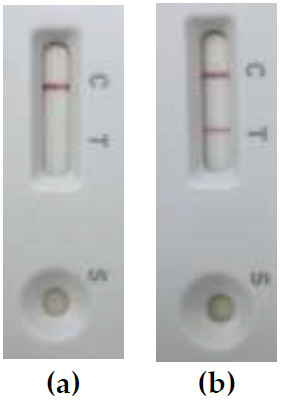
Results of the diphtheria toxin detection by LFIA in 6-hour Elek broth cultures inoculated with non-toxigenic *C. diphtheriae* NCTC 10356 ( **a**) and weakly toxigenic *C. diphtheriae* NCTC 3984 ( **b**) grown on Hoyle’s tellurite agar after plating of simulated diphtheria specimens.

The results of the LFIA study of 120 pharyngeal microbiota samples, spiked with a varied amount of test corynebacteria and grown on tellurite agar, completely concurred with the Tox status of the *C. diphtheriae*, *C. ulcerans* and *C. pseudodiphtheriticum* strains used for contamination of the samples. The LFIA was positive for all simulated samples loaded with toxigenic (by Elek test) strains and negative for the samples with non-toxigenic strains ( Table 2, Figure 5). False positive or false negative results were not observed.

The result of LFIA was recorded within 6 hours, and the diagnostic time from the moment of the clinical material inoculation to obtaining the result of the toxigenic corynebacteria presence/absence was 24–30 hours. Thus, using the LFIA-based approach, the clinical diagnosis of diphtheria can be confirmed or questioned by the end of the second working day from the start of the bacteriological study.

## Discussion and conclusions

It is well acknowledged, that diphtheria treatment is administered on the basis of clinical manifestations. But the onset of the disease, when it is easily amenable to therapy with diphtheria antitoxin, is virtually indistinguishable from widespread streptococcal acute tonsillitis. The clinical diagnosis of respiratory diphtheria in Europe is further complicated by the fact that infectious disease specialists rarely, if ever, encounter this disease. Accelerated and accurate laboratory confirmation of diphtheria diagnosis allows timely case management, which helps prevent complications or even death of the patient, as well as early counter-epidemic measures. Therefore, diagnostic laboratories need efficient procedures for rapid detection of DT-producing corynebacteria in clinical samples. Since taxonomic determination of the respective species of toxigenic corynebacteria is at first hand not urgently required for laboratory confirmation of a clinical diagnosis of diphtheria, we tested the following concise diagnostic algorithm for indication of toxigenic corynebacteria: 1) inoculation of a clinical sample on Hoyle’s tellurite agar, selective for corynebacteria; 2) examination of the Hoyle’s agar after 18–24 hours of incubation at 37°C for the presence of grey/black colonies resembling those of *C. diphtheriae/C. ulcerans*; 3) the study of these colonies for belonging to pathogenic corynebacteria, i. e. determination of their DT-producing capacity.

The abbreviated diagnostic approach – selection of suspicious colonies from the tellurite plate for toxigenicity testing by the Elek method – showed good results during the diphtheria epidemic in the countries of the former USSR in the 1990s. For this approach, diagnostic purified antitoxin and dried Elek medium have been developed and commercially produced in Russia ^14^. In Western countries, the Elek test, due to its sophisticated methodological requirements and in the absence of suitable ingredients, is performed mainly by specialized reference laboratories. Most diphtheria diagnostic laboratories identify and molecularly discriminate between toxigenic and non-toxigenic *C. diphtheriae* and *C. ulcerans* using PCR alone. However, there are no standardized commercial kits for targeting the genes of diphtheria agents. Besides, *C. diphtheriae* NTTB and *C. silvaticum* ^22^ strains with a non-expressed DT gene may occur, relativizing the diagnostic value of a positive *tox* PCR result.

The main test on which our version of the accelerated laboratory diagnosis of diphtheria is based is the newly developed immunochromatographic assay, LFIA. The high sensitivity and specificity of the test indicates the possibility of using it in place of the Elek test, which makes the detection of DT in bacterial cultures simpler, more standard and more reliable. LFIA allows one to quickly (within 6 hours after the appearance of suspicious colonies on Hoyle’s agar) identify DT-producing corynebacteria in a patient, thereby confirming the clinical diagnosis of diphtheria.

An indication of the ability of bacteria cultured from the patient to express the toxin is a necessary and sufficient condition for laboratory confirmation of the diphtheria diagnosis. However, clinical bacteriologists would probably benefit from a rapid and simple test for verification of the diphtheria corynebacteria grown on the plate of primary inoculation. Bacteria of the diphtheria group – *C. diphtheriae*, *C. ulcerans* and *C. pseudotuberculosis*, in addition to the ability to carry a functional *tox* gene, have some unique biochemical features, such as the absence of pyrazinamidase and the presence of cystinase activity. When comparing these two characteristics, the advantage remains with the cystinase test. This is due to the fact that the pyrazinamidase test is negative in corynebacteria of the diphtheria group, while in non-diphtheria corynebacteria it is positive – they cleave pyrazinamide with the formation of dark-colored reaction products. Therefore, even a slight admixture of non-diphtheria corynebacteria ( *C. pseudodiphtheriticum, C. striatum, C. amycolatum*, etc.) will mask the presence of bacteria of the diphtheria group. Considering that the cystinase test in diphtheria corynebacteria is positive, then even with contamination by cystinase-negative non-diphtheria corynebacteria, the black color of the Pisu medium will indicate the presence of bacteria of the diphtheria group. Taking into account the 100% agreement of the results of the cystinase test with the taxonomic position of corynebacteria in pure culture and in simulated specimens in our study, as well as the same duration of the LFIA and the Pisu test - 6 hours, we can consider the possibility of jointly conducting these two tests, in particular, for laboratory staff, not with sufficient experience in diagnosing diphtheria. When using both LFIA and cystinase test, their results will match: LFIA(+) and cystinase(+) in the presence of toxigenic diphtheria corynebacteria in the sample, and in the absence of these: LFIA(-) and cystinase(-). The combination of LFIA(-) and cystinase(+) is detected only in non-toxigenic diphtheria corynebacteria, which rarely inhabit the pharynx and nose of healthy people.

LFIA results indicating the presence of pathogenic corynebacteria on tellurite agar can be confirmed by PCR detection of the DT gene. We conducted pilot testing of RT-PCR ^23^ with DNA extraction by 5-minute boiling of the bacterial culture - grey/black colonies from the primary inoculation medium - and obtained encouraging results. No PCR inhibition was observed, indicating that the simplified DNA extraction method was satisfactory and not refractory to amplification (data not shown).

Our results showed that the concomitant pharyngeal microbiota (non-diphtheria corynebacteria, staphylococci, streptococci), which sparsely grow on tellurite plates of primary inoculation and could have entered the colony mixture sample for testing, did not interfere with either the growth of the diphtheria agent or DT production in Elek broth, neither the cystinase test nor the detection of gene *tox*.

After performing the LFIA, the 24-hour primary inoculation plate must be incubated for another day to grow the colonies. The next day, LFIA is recommended to be conducted with single colonies, and in case of a repeated positive test result, isolated pure cultures should be transported to a diphtheria reference laboratory for complete identification of microorganisms and confirmation of the "point-of-care" laboratory diagnosis. Also, after 48 hours of incubation, colonies of slowly growing potentially pathogenic corynebacteria may appear on Hoyle’s plates ^10, 14^, with which the above diagnostic procedure should be performed. If there is no growth of suspicious colonies after 48 hours, the plates are discarded and the answer is given that diphtheria corynebacteria were not detected in the clinical sample.

Thus, a laboratory algorithm for diagnosing diphtheria is developed, which may render less time in issuing a result and could promote the testing be performed in laboratories closer to the patient. After appropriate validation on the material of patients with diphtheria, the proposed algorithm can be used for routine diphtheria laboratory diagnosis. For reasons of standardization and unification of the method, it would be highly desirable that both LFIA and Elek broth be produced in a commercially available kit. In the future, research to improve the laboratory diagnosis of diphtheria would probably be aimed at creating methods for detecting DT or RNA encoding it directly in clinical material.

## Ethics and consent

All samples used in the study were residual samples from routine diagnostics in the context of secondary use of biological material and without the possibility of inferring patient data. The study was performed as part of the duty of the Bavarian Health and Food Safety Authority based on the German Infection Protection Act (IfSG). Therefore, no ethics approval had to be obtained.

## Data Availability

BioProject: WGS raw data are stored at the short reads archive (SRA). Accesssion number PRJNA939168; https://identifiers.org/NCBI/bioproject:PRJNA939168 ^19^. BioSample: Descriptive data of the clinical isolate *C. ulcerans* KL1902. Accession no SAMN33456493. https://www.ncbi.nlm.nih.gov/biosample/?term=no+SAMN33456493 ^20^. Genbank: The extracted sequence of the *C. ulcerans* KL1902 Diphtheria Toxin gene. Accession number OQ548089; https://www.ncbi.nlm.nih.gov/nuccore/OQ548089 ^21^. Zenodo: Lateral flow immunoassay-based laboratory algorithm for rapid diagnosis of diphtheria https://doi.org/10.5281/zenodo.7827917 ^15^. This project contains the following files: Elek test and LFIA for the detection of DT in corynebacteria. Study of colonies on tellurite agar with Pisu test and LFIA. Elek broth preparation. Data are available under the terms of the Creative Commons Attribution 4.0 International license (CC-BY 4.0).
