# Supplementary material for: Lateral flow immunoassay-based laboratory algorithm for rapid diagnosis of diphtheria
Source: Open Res Eur. 2023 Apr 25;3:62. [Version 1] doi: 10.12688/openreseurope.15038.1 (PMC10445807; doi:10.12688/openreseurope.15038.1)
Supplement: Peer review history

# Peer review: Reviewer response for version 1

**DOI:** 10.21956/openreseurope.16259.r31254

**Published:** 2023-05-15

**Authors:** Louisy Sanches dos Santos Sant'Anna

**Affiliations:** 1 Department of Microbiology, Immunology and Parasitology, Universidade do Estado do Rio de Janeiro, Rio de Janeiro, State of Rio de Janeiro, Brazil

In the present manuscript, Vyacheslav and colleagues proposed a Lateral Flow Immunoassay (LFIA)-based laboratory algorithm for the diagnosis of diphtheria, an acute infectious disease, preventable by vaccination, that has affected humans for hundreds of years and whose main symptoms result from the production of a potent and fatal exotoxin, the diphtheria toxin (DT), by the causative microorganism.

 The main etiological agent of diphtheria is *Corynebacterium diphtheriae*, however, in the last decades other closely related species of *Corynebacterium* genus have been found capable of harbour de DT gene ( *tox*). Since some of these species can be transmitted from animals, such as dogs and cats, diphtheria has been increasing in relevance in many countries. In addition, diphtheria has been considered to have a high potential for resurgence due to increasing vaccine hesitancy and migration movements, particularly from countries with high numbers of reported diphtheria cases.

 In this context, continuous surveillance and the development of faster, low-cost, and more sensitive laboratory diagnostic tests are needed for better diphtheria control. Thus, the work developed by Vyacheslav and colleagues is relevant and should be presented to the science community.

 Regarding to the manuscript, the Introduction section presented the research topic clearly, summarizing previous studies and emphasizing the relevance of this study for the field. Similarly, the Methods section was written clearly, providing sufficient information for replication in other studies. The results were also detailed and presented in a logical sequence. In the Discussion and Conclusions section, the authors presented the main findings of the study, which were interpreted in an unbiased manner and in the context of current and pertinent literature. Finally, I suggest a minor revision to improve the manuscript for publication. My comments are the following:

1. In the fourth paragraph of the introduction, the authors wrote: “…the major virulence factor in pathogenic corynebacteria.”
  The genus *Corynebacterium* comprises several pathogenic bacteria and many of them do not produce the diphtheria toxin but present other virulence factors, such as phospholipase D of *C. ulcerans* and *C. pseudotuberculosis*, I strongly suggest that the author revise this sentence. Similarly, I suggest that the terms “pathogenic corynebacteria” in the first paragraph of Discussion and Conclusion section be replaced by “diphtheria group corynebacteria”.
2. In the second paragraph of LFIA validation topic, the authors cited the strain NCTC 10356, identified as *C. diphtheria*e biovar belfanti. However, strains of this biovar were recently recognized as belonging to the species *C. belfantii* or *C. rouxii*. I suggest that the authors investigate the taxonomic position of the strain and, if possible, of other strains of the same biovar used in this work.
  Still, regarding the strains used and described in Table 1, I suggest that the authors detail the biovars of the strains of *C. diphtheria*e *tox*+ and *tox*- since the biovar belfanti was highlighted in the table.
3. Some acronyms (for example DT, LFIA, NTTB) have been used in Tables and Figures. I suggest that the cited acronyms be described in the illustrations. In addition, some of them were used in the text but were not described (for example PBS).
4. The countries of origin of some brands (Oxoid and Becton Dickinson) are missing.
5. Bacterial count values are not properly formatted in some parts of the Methods section (for example 10^5 cells per mL). In addition, authors must choose to use ml or mL.
6. The gene *tox* must be italicized in the fourth paragraph of the Results section.
7. In the sixth paragraph of Discussion and conclusions section, the authors used the acronym “RT-PCR”, but “RT” means reverse transcription and not real-time.

Is the study design appropriate and does the work have academic merit?

Yes

Is the work clearly and accurately presented and does it cite the current literature?

Yes

If applicable, is the statistical analysis and its interpretation appropriate?

Not applicable

Are all the source data underlying the results available to ensure full reproducibility?

Yes

Are the conclusions drawn adequately supported by the results?

Yes

Are sufficient details of methods and analysis provided to allow replication by others?

Yes

Reviewer Expertise:

Diphtheria surveillance and diagnosis; Corynebacterium diphtheriae and other Corynebacterium spp.: virulence, epidemiology, taxonomy and genomics

I confirm that I have read this submission and believe that I have an appropriate level of expertise to confirm that it is of an acceptable scientific standard.

# Response

**Published:** 2023-05-16

**Authors:** Vyacheslav Melnikov

**Affiliations:** Bavarian Health and Food Safety Authority, Germany

I thank Professor Sanches from the bottom of my heart for the detailed analysis of our publication. I appreciate and agree with all her statements and suggestions. I have now a great desire to propose a toast: "To diphtheria!". This sounds paradoxical at first glance. But there is nothing paradoxical in this. First, you should be very respectful of this infection. As rightly pointed out by Prof. Sanches, it has been killing and crippling people for centuries, so a frivolous attitude towards the disease is unacceptable. This is not a "forgotten" disease - last year diphtheria reached and spread across Europe, hiding in it. You should treat this Lady with reverence and pay attention to her constantly, and not from case to case. Western medicine often sins last: "It doesn't touch us, so let's leave everything as it is, we need money for more important matters - Covid, AIDS, hepatitis, tuberculosis…". Or: "Vaccinate everyone and you will all be happy, no matter what you instill and how effective this vaccine is - it now works and that's the question". No, you can't do that with diphtheria. It is a cyclic disease and it will always find a GAP and avenge the disrespectful attitude towards it! For example, it would seem, for no apparent reason, diphtheria became a ZOONOSIS and began to be actively spread by animals, including pets - cats and dogs. Its diphtheria toxin has undergone many changes, and whether diphtheria vaccination will be able to form reliable protection against "ulceraphtheria" or not is a big question that has risen in our queue for study. When the situation with diphtheria aggravates, as Prof. Gladysheva, all those who previously dealt with other infections, by magic, change their orientation, rushing to save the world from diphtheria, and pseudoscientific theories appear like mushrooms after the rain, that put sticks in the wheels of real specialists. Therefore, everything must be done on time. As my great-grandfather, a Russian Professor of Medicine Dr. Makarov, from whom our medical dynasty began, and who was shot by Stalin's thugs in 1937 as a "Japanese spy" (for operating on a Japanese consul) used to say: "One gram of prevention is better than a kilogram of therapy". And the second. Diphtheria gathered at one table caring people, researchers, excellent professionally and personally, who support each other with an open soul and kind heart, but at the same time do not compromise their principles: "Plato is my friend, but the truth is dearer!". Thanks again Prof. Sanches for her selfless service to science, and, in her person, for the Brazilian School of Microbiology, rich in traditions and achievements, widely known and respected in the world.

# Peer review: Reviewer response for version 1

**DOI:** 10.21956/openreseurope.16259.r31257

**Published:** 2023-05-09

**Authors:** Sylvain Brisse

**Affiliations:** 1 Biodiversity and Epidemiology of Bacterial Pathogens, Université Paris Cité, Institut Pasteur, Paris, Île-de-France, France

Diphtheria was a major killer of children before the implementation of large-scale vaccination, which has enabled to largely control this disease. As a result of its virtual disappearance in high-income countries, diphtheria became a largely neglected disease, even though it still causes endemic disease and outbreaks in multiple world regions. Early administration of diphtheria antitoxin is critical to limit the detrimental effects of the diphtheria toxin during infection; however, antitoxin use can have severe undesired effects and should be ideally used only in patients with confirmed diphtheria. Therefore, rapid and highly specific diagnostic is critical, and because the clinical presentation is not fully specific, laboratory procedures are absolutely required. Here, the authors present the development and laboratory evaluation of a novel diphtheria diagnostic assay, which enables the detection of diphtheria toxin production more simply and more rapidly than currently existing methods.

 The diphtheria toxin (DT) consists of the assemblage of two fragments, which result from the nicking of the precursor protein expressed from a single coding sequence, the *tox* gene. The present work builds on previous work by the same group, in which two monoclonal antibodies that target distinct (yet undefined) epitopes of the fragment B were selected and used in an ELISA format. Here, these antibodies are used in a lateral flow immunoassay (LFIA) format, which simplifies the laboratory procedure. While one antibody is used for capture on the solid support, the other antibody is colloidal gold-labelled and used for detection. In this work, the novel test was applied to 154 previously characterized toxigenic isolates and 151 non-toxigenic ones, belonging to the diphtheria group *Corynebacteria*. While no false positive was detected, one false-negative result was found for a *Corynebacterium ulcerans* isolate with a polymorphism in the DT fragment B. By using spiked human pharyngeal samples, the authors additionally show the applicability of the method directly from colonies that have grown on the selective agar plate used for the primary isolation from the clinical sample, thus saving the extra time necessary for purification of the colonies through subsequent subcultures. However, attempts to detect the toxin production by directly inoculating liquid broth with the clinical specimen, were unsuccessful, implying a need for the 18-24 hours selective medium agar step. The authors further indicate that the diagnostic based on DT detection can be complemented by the identification of *Corynebacteria *of the diphtheria group by a cystinase test.

 It is important to contextualize the present work in the broader context of the various approaches to the diagnostic of diphtheria. The diagnostic of diphtheria is complexified by heterogeneous definitions of diphtheria itself. The classical definition focuses on toxin-producing *Corynebacterium diphtheriae* in respiratory infections (with the late addition of toxigenic *Corynebacterium ulcerans*, which is transmitted to humans from animals such as dogs and cats, but produces a very similar toxin). For practical reasons, and because the causative organisms can be associated with cutaneous on invasive infections, less strict definitions of diphtheria are in use for surveillance purposes in some countries. An alternative definition considers the presence of the *tox* gene itself, rather than the production of the diphtheria toxin. This definition is linked to the widespread use of PCR methods for diagnostic, as these are practical to implement and can be very rapid. Further, PCR detection can be applied directly on clinical specimens, saving the culture time and needed infrastructure.

 So, if diagnostic PCR is reliable and rapid, why spend efforts on DT production detection? As the authors describe, non-toxigenic toxin-gene bearing isolates exist (and were, as it happens, initially described by the first author of the present work). These isolates do not induce the clinical symptoms associated with the toxin and there is no justification that the infections they cause should be managed with antitoxin. When using *tox* PCR-based diphtheria diagnostic methods, NTTB isolates would thus represent false positives. The detection of DT production, rather than tox gene presence, has the merit to avoid these false positive cases. But what is the magnitude of this problem? In large scale surveillance cohorts from the UK and France, the NTTB typically represented 10 to 20% of *C. diphtheriae* isolates, which is far from negligible. Of note, novel approaches such as rapid DNA sequencing of the *tox* gene, represent interesting avenues to address the issue of NTTB detection by molecular methods.

 The detection of diphtheria toxin using antibodies may be affected by toxin gene variation in the natural populations of the target bacteria. This was illustrated by the false negative result obtained by the authors with one *C. ulcerans* isolate with a variant toxin gene. An important unknown at this stage is the ability of the proposed test to detect all known variants of the toxin gene. Here, the *tox* gene diversity of the sample used to evaluate the test was not described, even though it can be expected to be large, given that it comprises 154 toxigenic isolates obtained in the German reference laboratory over more than 10 years from humans and animals, and belonging to different species of the diphtheria group. However, diphtheria toxin variants that may escape detection might be prevalent in other world regions, and this issue should be carefully evaluated in future work.

 Compared to the Elek test, which is the current standard for DT detection, the novel method is much simpler to perform. Its main advantage may therefore be to enable diphtheria diagnostic in remote settings, not equipped with PCR diagnostic nor with the Elek test. While such approach represents a clear advance, one major caveat remains: the availability of the test. It is to be hoped that the technological advances in diphtheria diagnostic, such as the one presented here, combined with the re-emergence of diphtheria in several world regions, as also observed in Europe in 2022, will stimulate the sustainable production and wide availability of diphtheria diagnostics where they are most needed.

Is the study design appropriate and does the work have academic merit?

Yes

Is the work clearly and accurately presented and does it cite the current literature?

Yes

If applicable, is the statistical analysis and its interpretation appropriate?

Not applicable

Are all the source data underlying the results available to ensure full reproducibility?

No source data required

Are the conclusions drawn adequately supported by the results?

Yes

Are sufficient details of methods and analysis provided to allow replication by others?

Partly

Reviewer Expertise:

Diphtheria population biology, surveillance, diagnostic, genomics, evolution

I confirm that I have read this submission and believe that I have an appropriate level of expertise to confirm that it is of an acceptable scientific standard.

# Response

**Published:** 2023-05-12

**Authors:** Vyacheslav Melnikov

**Affiliations:** Bavarian Health and Food Safety Authority, Germany

I am very grateful to Professor Brisse for his scrupulous reviewing our publication. In fact, his review can be published as an editorial to a collection of articles on the current state of diphtheria research. In fact, we all have pained to discuss the burning issues of a clear increase in the incidence of diphtheria in Europe and the world, and to come to a common denominator on the main problems. We need a good shake to turn over the accumulated ancient diphtheria papyri and separate the wheat from the chaff. Otherwise, soon at a lecture on diphtheria, we will not be able to say anything intelligible to representatives of student youth who are striving to get to the bottom of the truth. I would like to add my "five cents" to the flaring discussion. I think that the "old school" infectious disease specialists from Russia, where there was a diphtheria epidemic in the 1990s, India, Brazil and other countries that are constantly dealing with this infection, will agree with me that our banner must have written in indelible ink in large print: RESPIRATORY DIPHTHERIA IS CAUSED EXCLUSIVELY BY TOXIGENIC STRAINS OF *Corynebacterium diphtheriae, *that is, those that produce the diphtheria toxin. Incidentally, *C. ulcerans*, even toxigenic, is not a very significant diphtheria agent, and does not attract attention (yet, I think) in high-rate diphtheria countries, since outbreaks of the disease caused by *C. ulcerans* are not observed, and cases of "ulceraphtheria" are rarely severe, possibly due to low production of the toxin [ https://www.mdpi.com/2075-4418/12/9/2204]. Cutaneous diphtheria caused by toxigenic *C. diphtheriae *also proceeds (for some unclear reasons) mainly without a lethal outcome or complications. Therefore, I agree with Prof. Brisse, that there are many issues in diphtheria terminology that we need to resolve through joint efforts. And yet, in every diphtheria diagnostic manual one will find the phrase that a laboratory diagnosis of diphtheria cannot be established only on the basis of PCR. PCR can only confirm the result of the detection of His Majesty TOXIN (currently using the good old but complex and inaccurate Elek test). La diphtérie est étroitement liée à la toxine diphtérique. The toxin causes main symptoms of the disease, which are stopped exclusively by antitoxin and prevented by toxoid. Accordingly, the causative agent of diphtheria at the crime scene can only be caught by "fingerprints" of its toxin, if, of course, you want your testimony to be accepted in court. I have no doubts about the diagnostic capabilities of PCR. But is it always possible to trust the results of toxin gene detection in the clinical material in the absence of field-tested PCR kits on the market? I'm not even talking about the lack of PCR laboratories within walking distance from diphtheria foci. And dragging samples for PCR to distant lands is a thankless task. Now, if someone would take up the development of a standard portable device for PCR diagnostics of diphtheria and train Médecins Sans Frontières!

 Regarding the strain *C. ulcerans *KL 1902, we still do not know if it is pathogenic. It is possible that the mutation that made the strain LFIA-invisible causes critical changes in the structure of the toxin that make it non-toxic. This is for us to check. In addition, an active mutation process in the gene *tox* can disrupt not only the binding of monoclonal antibodies to the toxin molecule, but also the binding of primers to DNA, thereby making such toxigenic strains invisible to PCR. As a result, it must be said that any clinical bacteriologist considers it his duty to “hold the microbe in his hands” in order to identify it, to check its sensitivity to antibiotics and molecular type. In general, no matter what modern toxin or its gene detection methods are used to diagnose diphtheria, we, classical microbiologists who pay tribute to Pasteur, Koch, Metchnikoff, are still indispensable. I share Professor Brisse’s regrets about the lack of diphtheria LFIA on the market. But I hope that the research carried out by us will encourage investors to breathe life into the LFIA, a test that was inspired dozens of years ago by Izabella Mazurova, Kathy Engler and Androulla Efstratiou [ https://pubmed.ncbi.nlm.nih.gov/2508377/, https://pubmed.ncbi.nlm.nih.gov/11773096/].

 Sincerely, V. Melnikov

# Peer review: Reviewer response for version 1

**DOI:** 10.21956/openreseurope.16259.r31258

**Published:** 2023-05-05

**Authors:** Irina Gladysheva

**Affiliations:** 1 Laboratory of Biomedical Technologies, Institute for Cellular and Intracellular Symbiosis UrB RAS, Orenburg, Russian Federation

Title and authors

**Lateral flow immunoassay-based laboratory algorithm for rapid diagnosis of diphtheria**

 Vyacheslav G. Melnikov, Anja Berger, Alexandra Dangel and Andreas Sing.

Summary

 I am pleased to assess the manuscript in Open Research Europe. In this manuscript, the authors describe an LFIA-based diphtheria laboratory diagnostic algorithm that can accelerate and improve the accuracy of diphtheria diagnosis in the world.

 When I give lectures to practical bacteriologists I can often hear: “Why are the ‘ancient’, labor- and resource-intensive methods of diagnosing diphtheria still being used in the era of -omics, and a simple test has not yet been developed?” When there is an epidemic of some forgotten disease, everyone rushes to invent rapid diagnostic tools. But it's usually too late. Work should be done in advance in order to better prepare for the epidemic: “A stitch in time saves nine”! The article of Melnikov *et al.* is an example of work done in advance.

 The deep conviction that diphtheria, thanks to mass vaccination, has practically disappeared, does not currently find its confirmation. Geopolitical changes lead to population migration and diphtheria outbreaks, as well as the spread of other deadly infections in various parts of the world. The recent increase in the incidence of diphtheria in India, Ethiopia, Nigeria, Bangladesh, Yemen, Venezuela and even Europe underline the need for renewed efforts not only to better understand the pathogenesis of the disease, but also necessitate its rapid diagnosis for prompt and timely treatment and anti-epidemic measures. Currently, the diagnosis of diphtheria, based on the detection of diphtheria toxin by agar immunoprecipitation or the Elek test, is complex and laborious. The new diagnostic technology proposed by the authors and based on lateral flow immunoassay (LFIA) is very relevant and promising. To my mind, for the first time in the last 70 years, it has been proposed to significantly simplify and speed up the diagnosis of diphtheria, making it accessible even to small local laboratories. This accelerated approach proved successful during the diphtheria epidemic in Russia (1991-2000). The authors of the manuscript have updated it with the LFIA on monoclonal antibodies. Once the LFIA-based approach has been tested on material from patients with diphtheria, it can be recommended to a wide range of bacteriological laboratories conducting diphtheria diagnosis, which will allow diphtheria to be diagnosed at the patient's bedside, rather than sending clinical material to the WHO laboratory in London for diagnosis.

 The evaluation of the new diagnostic methodology was carried out on 322 strains of corynebacteria and 360 simulated diphtheria samples, all experiments were carried out in triplicate, which confirms the reliability of the study. The authors described the methods, results and discussion of the study in sufficient detail. In my opinion, the work is original and represents new data. The article can be accepted in its current form.

Is the study design appropriate and does the work have academic merit?

Yes

Is the work clearly and accurately presented and does it cite the current literature?

Yes

If applicable, is the statistical analysis and its interpretation appropriate?

Yes

Are all the source data underlying the results available to ensure full reproducibility?

Yes

Are the conclusions drawn adequately supported by the results?

Yes

Are sufficient details of methods and analysis provided to allow replication by others?

Yes

Reviewer Expertise:

Corynebacterium spp., interbacterial interactions, antimicrobial activity, antioxidant activity, secondary metabolites

I confirm that I have read this submission and believe that I have an appropriate level of expertise to confirm that it is of an acceptable scientific standard.

# Response

**Published:** 2023-05-05

**Authors:** Vyacheslav Melnikov

**Affiliations:** Bavarian Health and Food Safety Authority, Germany

Dear Professor Gladysheva,

 Thank you for your time and deep dive into this material. It is great to hear words of encouragement from the country where I trained as a medical doctor and microbiologist, started my scientific career, and was involved in the fight against a major diphtheria epidemic in the early 1990s. I am aware of your work on C *orynebacteria *and looking forward to cooperation in the future! Sincerely,

 Vyacheslav Melnikov
